# Acquired von Willebrand Disease Associated with Mantle Cell Lymphoma

**DOI:** 10.1155/2018/7973297

**Published:** 2018-01-30

**Authors:** Dominique Maas, Britta Laros-van Gorkom, Sanne Gianotten, Marjan Cruijsen, Waander van Heerde, Marten Nijziel

**Affiliations:** ^1^Department of Hematology, Radboud University Medical Center, Nijmegen, Netherlands; ^2^Hemophilia Treatment Center, Radboud University Medical Center, Nijmegen, Netherlands; ^3^Department of Laboratory Medicine, Laboratory for Hematology, Radboud University Medical Center, Nijmegen, Netherlands

## Abstract

We present a rare case of acquired von Willebrand syndrome (AVWS) caused by a mantle cell lymphoma. A 61-year-old male suffered from recurrent bleeding symptoms since a few months. Initially, physical examination was normal. von Willebrand factor antigen (VWF:Ag) level and factor VIII activity (FVIII:C) were low (0.31 IU/ml and 0.43 IU/ml, resp.). Ristocetin cofactor activity (VWF:RCo) was 0.09 IU/ml, and collagen binding activity (VWF:CB) was 0.10 IU/ml. VWF:RCo/VWF:Ag ratio was 0.29, and RIPA value was normal. Highest molecular weight VWF multimers were absent. A diagnosis of von Willebrand Disease (VWD) type 2A was made. However, no genetic mutation was found. No inhibitory antibodies against VWF or factor VIII were detected. A few months later, cervical, axillary, and inguinal lymphadenopathy was found on physical examination. A CT scan confirmed multiple enlarged lymph nodes, and a clonal B-cell population matching a mantle cell lymphoma was detected in the bone marrow. Chemoimmunotherapy resulted in a very good partial remission and concomitantly in a rapid decrease of bleeding problems and complete normalization of FVIII:C and VWF:Ag. The diagnosis of AVWS cannot be rejected by negative mixing studies due to difficulties in the detection of autoantibodies and because of a highly heterogeneous pathogenesis of AVWS. When the suspicion of AVWS is high, an extensive investigation should be performed to find the underlying cause.

## 1. Introduction

Acquired von Willebrand syndrome (AVWS) is a rare bleeding disorder, associated with a variety of underlying medical conditions. Due to similar clinical and laboratory manifestations, differentiation of congenital and acquired von Willebrand Disease (VWD) can be challenging. A negative family history and onset of bleeding symptoms later in life make an AVWS more likely than a congenital VWD [1]. When the suspicion of AVWS is high, a search for the underlying cause should be initiated. We present a rare case of AVWS associated with a mantle cell lymphoma.

## 2. Case Presentation

A 61-year-old man was referred to our department for evaluation of a hemorrhagic diathesis. He suffered from recurrent epistaxis, hematuria, spontaneous hematomas, and rectal bleeding since a few months. In 2003, he experienced a massive bleeding after a transurethral resection of the prostate due to surgical reasons. In the following years, he underwent multiple surgical procedures without bleeding complications. Family history was negative for bleeding disorders, although his father died from an intestinal bleeding without a previous history of bleeding diathesis. Besides a few hematomas on his arms and hands, physical examination was normal. Laboratory studies showed a normal platelet count (255 × 10^9^/l, reference range 150–400 × 10^9^/l), a normal prothrombin time of 13 seconds (INR 1.0), and a slightly lengthened activated partial thromboplastin time (APTT) of 35 seconds (reference range 26–34 seconds). Closure times obtained by the PFA-100 (platelet function analyzer) were prolonged for both the collagen-epinephrine cartridge (>300 seconds, reference range <170 seconds) and the collagen-adenosine diphosphate cartridge (>269 seconds, reference range <120 seconds). Further studies revealed a low FVIII:C (0.43 IU/ml, reference range 0.60–1.50 IU/ml) and VWF:Ag level (0.31 IU/ml, reference range 0.50–2.10 IU/ml). VWF:RCo was 0.09 IU/ml (reference range 0.40–2.10 IU/ml), and VWF:CB was 0.10 IU/ml (reference range 0.57–1.79 IU/ml). VWF:RCo/VWF:Ag ratio was 0.29 (reference range >0.7), and RIPA value was normal. The highest molecular weight VWF multimers were absent.

A diagnosis of VWD type 2A was made, but no genetic mutation was identified by polymerase chain reaction coupled with Sanger sequencing. A desmopressin (DDAVP) infusion test was not done because of previous arrhythmias. He did not use any anticoagulants.

Tranexamic acid and Haemate P had little effect on bleeding symptoms and factor VIII activity. Because of persistent hematuria, treatment consisting of 3,000 units of Haemate P twice a day was started. Ninety minutes after infusion of 3,000 units of Haemate P, FVIII:C was 0.53 IU/ml. Higher doses of Haemate P (4,000 units, twice a day) were required. Eventually, bleeding symptoms resolved. The clinical suspicion of an AVWS was high, and mixing tests were done. APTT on a plasma sample was measured before and after incubation at 37°C for 120 minutes. However, no inhibitory antibodies directed against VWF or factor VIII were detected.

A few months later, the patient complained of increased rectal bleeding and daily epistaxis. Physical examination showed slightly enlarged cervical, axillary, and inguinal lymph nodes. FVIII:C was 0.07 IU/ml and VWF:Ag 0.22 IU/ml. Again, mixing tests were performed, but no autoantibodies were identified. A CT scan confirmed multiple enlarged lymph nodes. A bone marrow biopsy was carried out. Multiple nodular lesions composed of small atypical lymphoid cells with irregular nuclei and micronucleoli were detected. Immunohistochemical study demonstrated that the lymphoid cells were positive for CD5, CD20, CD79a, and cyclin D1 and negative for CD3, CD138, and CD23. Fluorescence in situ hybridization showed translocations of chromosome 11q13. Both surgeon and radiologist were not able to biopsy a lymph node. Treatment with R-CHOP chemoimmunotherapy resulted in a very good partial response. A second bone marrow biopsy was done, and only a few cyclin D1-positive cells were detected. A rapid disappearance of the bleeding problems was observed. FVIII:C and VWF:Ag completely normalized.  Table 1 summarizes the exact laboratory values before, during, and after therapy.

## 3. Discussion

AVWS is a rare hemorrhagic disorder. Multiple underlying causes have been reported, mainly lymphoproliferative, cardiovascular, myeloproliferative, and autoimmune disorders and neoplasia [1, 2]. Our patient was diagnosed with a mantle cell lymphoma, a rare subtype of B-cell non-Hodgkin lymphoma. The relationship between AVWS and non-Hodgkin lymphoma has earlier been described in several case reports, mainly in patients with a mucosa-associated lymphoid tissue (MALT) lymphoma [3, 4]. To our best knowledge, the association between AVWS and mantle cell lymphoma was never reported previously.

The pathophysiology of AVWS is highly heterogeneous and multifactorial. Several mechanisms have been reported since the description of the first case of AVWS in 1968. Frequently proposed mechanisms are development of specific or nonspecific autoantibodies directed against VWF, adsorption of VWF by malignant cell clones, and increased proteolytic degradation of VWF under conditions of high shear stress. Rarely, synthesis of VWF is reduced, particularly in patients with hypothyroidism [5].

In patients with lymphoproliferative disorders, AVWS is mainly caused by the formation of autoantibodies directed against VWF [5]. A functional defect of plasma VWF is induced by binding of these specific inhibitors to functional epitopes of VWF. Secondly, nonneutralizing autoantibodies binding to nonfunctional domains of VWF can accelerate the elimination of the large VWF multimers from the circulation by the formation of immune complexes [2, 5]. In our patient diagnosed with a mantle cell lymphoma, no inhibitory antibodies were detected. Such a negative test result does, nevertheless, not reject the diagnosis of AVWS. The detection rate of inhibitors in AVWS is low [1, 5]. Inhibitory activity against FVIII or VWF was confirmed by mixing tests in only 25% of patients with AVWS associated with a lymphoproliferative disorder [2]. However, autoantibodies may still be present in the circulation despite a negative test result [5]. Detection can be prevented due to saturation of inhibitors in immune complexes. Measurable functions of VWF will not be inhibited when the clearance of VWF from the circulation is increased by nonneutralizing autoantibodies. Enzyme-linked immunosorbent assays (ELISAs) can be used to detect these antibodies, but these assays are prone to false-positive results due to the presence of ABO blood group antigens in immobilized plasma-derived VWF. Standardized assays are not yet available [1, 5].

Furthermore, other pathogenic mechanisms may be responsible for the occurrence of AVWS in our patient. Selective adsorption of VWF onto malignant cells or other cell surfaces has been described in patients with AVWS associated with lymphomas. In a patient with a marginal zone lymphoma and AVWS, flow cytometry revealed that the neoplastic lymphocytes strongly expressed platelet glycoprotein Ib and VWF [6].

Selective adsorption of the VWF protein by tumour cells was also reported in patients with multiple myeloma [7], Waldenström's macroglobulinemia [8], and adrenal cortical carcinoma [9].

Standard therapies for inherited VWD often have little effect in patients with AVWS. Moreover, the bleeding phenotype of patients with AVWS associated with lymphoproliferative disorders is generally more severe than in patients with AVWS caused by other underlying diseases [5]. Symptomatic treatment with for instance desmopressin, VWF-containing concentrates, antifibrinolytics, intravenous immunoglobulins, or plasmapheresis can be initiated in case of acute bleeding episodes [1]. Management of the underlying disorder can lead to long-term remission of the acquired coagulopathy and should therefore be considered whenever possible [1].

In the Netherlands, primary treatment for mantle cell lymphoma consists of three courses of R-CHOP chemoimmunotherapy and two courses of high-dose cytarabine followed by autologous stem-cell transplantation. In our patient, plasma levels of VWF:Ag improved gradually and completely normalized after three cycles of chemotherapy. Bleeding symptoms resolved completely.

Resolution of AVWS after initiation of therapy has indeed been described in several patients with a lymphoma [3, 4]. However, a case of AVWS in a patient with a small lymphocytic lymphoma illustrates that successful management of the underlying condition not always results in remission of the coagulopathy [10].

In conclusion, we described a rare case of a patient with AVWS caused by a mantle cell lymphoma. Initiation of the appropriate treatment with chemoimmunotherapy completely corrected the acquired coagulopathy. Because of difficulties in the detection of autoantibodies and the fact that different mechanisms contribute to the pathogenesis of AVWS, negative mixing studies do not reject the diagnosis of AVWS. Suspicion of AVWS is raised by onset of bleeding symptoms later in life and a negative bleeding history. A thorough work-up to find the underlying cause is recommended strongly.

## Figures and Tables

**Table 1:** Laboratory values before, during, and after therapy.

	FVIII:C (IU/ml)	VWF:Ag (IU/ml)	VWF:RCo (IU/ml)
Normal values	0.60–1.50	0.50–2.10	0.40–2.10
Prechemotherapy	0.34	0.32	0.31
After R-CHOP cycle 1	0.60	0.51	n/a
After R-CHOP cycle 2	2.18	1.47	>1.00
After R-CHOP cycle 3	2.12	1.70	>1.00
After cytarabine + rituximab (cycles 1 and 2)	2.18	1.94	n/a
Follow-up	2.32	2.03	>1.00

n/a: not available.
